# The intriguing world of archaeal viruses

**DOI:** 10.1371/journal.ppat.1008574

**Published:** 2020-08-13

**Authors:** Jennifer Wirth, Mark Young

**Affiliations:** 1 Plant Science and Plant Pathology, Montana State University, Bozeman, Montana, United States of America; 2 Department of Microbiology and Immunology, Montana State University, Bozeman, Montana, United States of America; University of Michigan Medical School, UNITED STATES

Viruses are among the most abundant biological entities on earth, outnumbering cells in some environments by more than an order of magnitude. Viruses of Archaea (termed archaeal viruses) are some of the most unusual and least understood group of viruses. However, even with our limited knowledge of these remarkable viruses, their characterization has led to major and sometimes startling discoveries. Major questions facing the field include the following (Fig 1).

**Fig 1:**
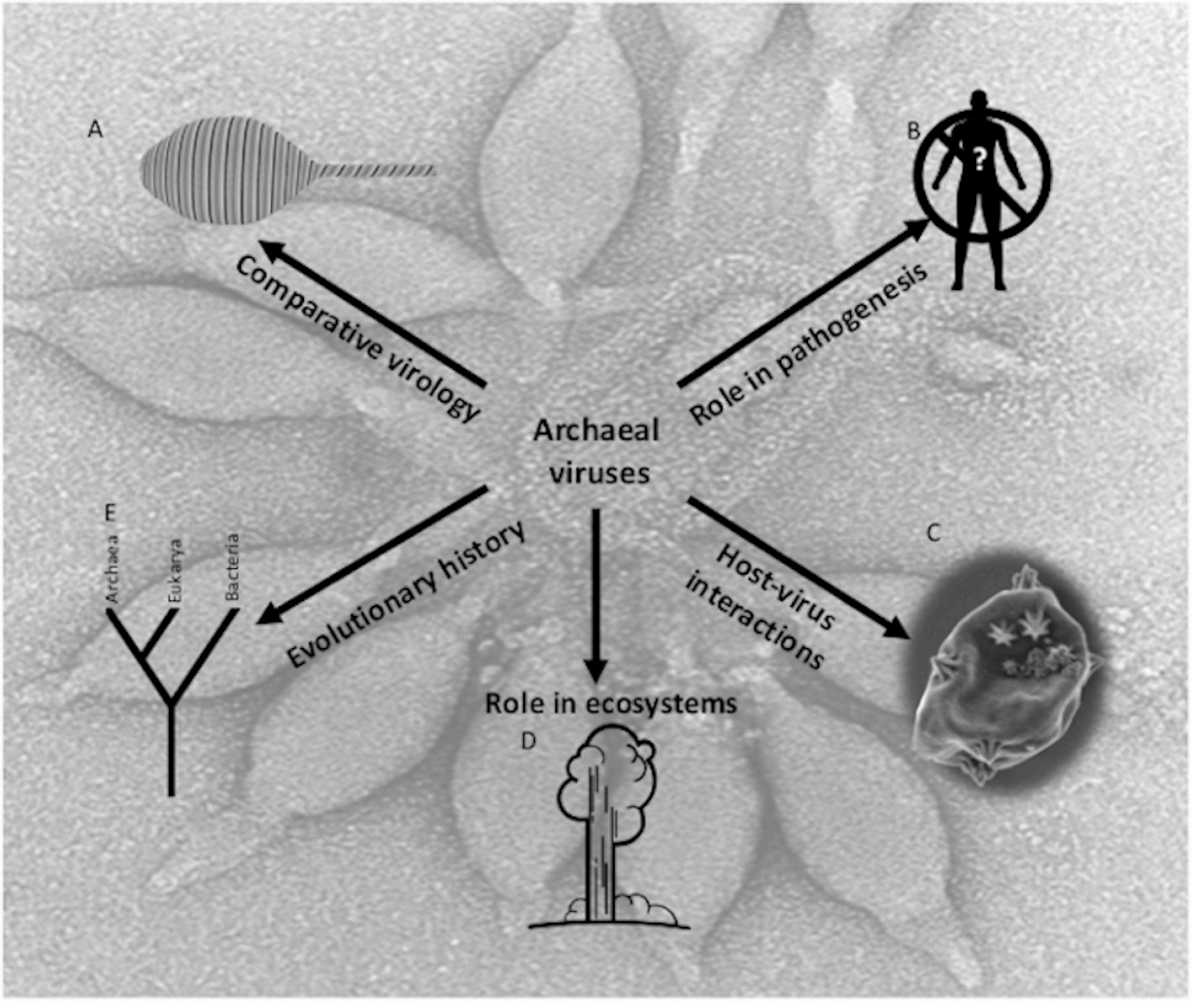
Interesting aspects of archaeal virology. (A) *Acidianus* tailed spindle virus schematic (and TEM background image) illustrates the unique morphologies that can be found among archaeal viruses. (B) Archaeal viruses and their hosts have no known role in human pathogenesis. (C) Archaeal viruses have revealed novel virus-host interactions, including a novel pyramid-based lysis mechanism. (D) Archaeal viruses are common but not limited to extreme environments. (E) The evolutionary history of archaeal viruses involves two tracts, one unique to Archaea and one with relationships to eukaryotic and bacterial viruses. *Image credits*: *Old Faithful by Nick Bluth from the Noun Project (D) and Evolutionary Tree by A Beale from the Noun Project (E)*.

## Are archaeal viruses different from viruses infecting eukaryotes and bacteria?

The answer to this question is both yes and no. Known archaeal viruses can be roughly divided into two categories: those that are morphologically and genetically unique to Archaea and those with clear structural and genetic homologs to both bacteriophage and eukaryotic viruses [1]. For example, many archaeal viruses infecting halophylic archaea resemble the tailed viruses belonging to the Caudovirales order of bacterial viruses. Archaea-specific viruses represent some of the most unique viruses known, from bottle-shaped viruses, to spindle-shaped viruses, to two-tailed viruses whose tails “grow” once they are released from infected cells. Some of these viruses assemble into previously unknown architectures. For example, *Acidianus* tailed spindle virus (ATSV) uses rope-like strands that slide against one other to assemble varying widths of its lemon-shaped capsid and tail, and packages the double stranded DNA (dsDNA) genome asymmetrically within capsid [2, 3]. Typically, the only identifiable viral gene homologs in most archaeal virus genomes are to other archaeal viruses or their cellular hosts, with few having known functions. Even though only 65 archaeal viruses are known, they comprise 17 new virus families based on their diverse virion morphologies and gene content [1]. Thus far, the majority of archaeal viruses have dsDNA linear or circular genomes, while two families have single stranded DNA genomes. No RNA viruses have been isolated, but they have been detected in environmental metagenomic studies [4]. Many of the unusual features of archaeal viruses that infect hyperthermophiles likely represent adaptations required for replication in their archaeal hosts and for stability in the extreme conditions found in high-temperature acidic hot springs. The majority of archaeal viruses have been isolated from only two of 14 (recognized and proposed) archaeal host phyla, making it highly likely that sampling of unexplored host phyla will only further expand our understanding of archaeal virus diversity.

## What diseases are associated with Archaea and their viruses?

Surprisingly, no Archaea is known to cause human disease and therefore no archaeal viruses are known to be involved with modulating pathogenesis in humans. Unlike bacterial pathogens, in which it is known that bacteriophage can contribute to pathogenesis, for example in the case of the cholera toxin, no such cases are known for archaeal viruses. Even though archaeal species are residents in the human oral cavity, human skin, and human gut, where they comprise up to 10% of the human gut anaerobic community, none are known to cause disease [5]. It is unclear why this is the case. One possibility is that it reflects a lack of direct studies that investigate the role of Archaea and their viruses in disease. However, in the age of abundant metagenomic DNA sequencing of disease states, this is becoming a less likely explanation. In contrast, it may reflect properties of the archaeal lifestyle that are not compatible with disease. The lack of clear evidence for pathogenicity of Archaea and associated archaeal viruses in humans is in itself an intriguing observation, which suggests a fundamentally different set of associations between humans, Archaea, and their viruses.

## What are unique features of archaeal virus-host interactions and viruses?

Studies of host-virus interactions in Archaea have led to new discoveries that expand our understanding of host-virus interactions in the viral world. Several archaeal viruses have emerged as model systems for examining host-virus interactions. These include the 72-nm spherical *Sulfolobus* turreted icosahedral virus 1 (STIV1), which packages a 17-kbp dsDNA genome [6], and the 23 × 900-nm *Sulfolobus islandicus* rod-shaped virus 2 (SIRV2), which packages a 35-kbp dsDNA genome [7]. Both of these lytic viruses share a new and highly unusual virus egress mechanism. Cells infected with these viruses form approximately 150-nm-tall seven-sided virus-associated pyramid (VAP) structures on their surfaces that open like the petals of a flower to rupture the cell’s external S-layer, providing a hole for virus egress [8, 9]. VAP formation is controlled by a single viral gene, which when expressed in bacterial and eukaryotic cells results in VAP formation [10]. While viruses with enveloped virions are found in all three domains life, a new lipid structure and likely a novel acquisition mechanism have been discovered in *Acidianus* filamentous virus 1 (AFV1) [11]. The AFV1 viral envelope acquired from the host cell takes on an unusual U-shaped conformation in the virion envelope that is not present in the host membrane from which it is derived. The never-before-seen U-shaped single monolayer lipids are created by the bending of the long flexible lipids present in the host membrane that are repurposed for creating the thinner AFV1 membrane envelope [11]. This structure likely allows for the high curvature found in the long thin filamentous virus capsid. How the virus accomplishes this feat is unknown. Interestingly, it has also been recently observed that several hyperthermophilic archaeal viruses from diverse virus groups, including SIRV and AFV, package their dsDNA genomes as the rarely seen in nature A-form DNA, as compared with more commonly found B-form DNA. In addition, viral proteins and virus genome features have been identified that modulate host-virus interactions, often to the benefit of the infected cell. This includes viral genomes carrying clusters of regularly interspaced short palindromic repeat (CRISPR) arrays, which likely prevent other viral infections within the same cell [12]. Furthermore, archaeal viruses are a source of potent anti-CRISPR proteins whose biochemical analysis is providing new insights into the CRISPR defense systems [13]. For example, it was recently shown that some archaeal viruses encode for effective anti-CRISPR proteins that are also found in some bacteriophage and plasmids [14].

## What role do archaeal viruses play in ecosystems?

Determining the range of ecological roles viruses play in natural environments is a central question driving studies in environmental virology today. Acidic hot springs found in Yellowstone National Park provide ideal environments for such studies because they consist of simplified microbial communities made up principally of archaeal hosts and their viruses. This allows for archaeal host-virus interactions to be studied in their natural setting in the absence of other microbial players. For example, one studied Yellowstone hot spring (NL01) consists of 7–8 archaeal host cell types (average nucleotide identity >95% in single-cell assembled genomes with >30% coverage) supporting the replication of 115 archaeal viruses. In this hot spring, we have learned that most if not all cells are constantly interacting with viruses [15]. We have begun to appreciate that virus-virus competition for potential host cells is almost as important as host-virus interactions. We are also discovering that a wide range of host-virus associations can occur, from pathogenic lytic infections to beneficial chronic infections. The high prevalence of chronic infections in Archaea may be a mechanism whereby a chronically infected cell pays a lower price for the protection provided against another more pathogenic virus that might otherwise kill the cell [15]. Furthermore, low cell density environments of hot springs may favor chronic viruses propagated by vertical transmission while viruses using horizontal transmission strategies may not succeed in finding new hosts [16]. Recent work examining deep marine sediments has identified that the impact of viruses may be greater on the archaeal members of the community than on the bacteria [17]. This work demonstrated that archaeal cells are approximately equal to bacterial cells in deep sea sediments. However, virus-mediated archaeal cell lysis occurs at a higher proportion than for bacterial cells, and thus archaeal viruses may be major driver of biogeochemical cycling in the oceans [17].

## Is the evolutionary history of archaeal viruses different from that of other viruses?

In general, deciphering the evolutional history of viruses is a difficult task due to the lack of universally shared viral genes and the presence of extensive horizonal gene transfer among viruses and between viruses and their hosts. It is particularly difficult with archaeal viruses because we only have limited knowledge of archaeal virus genomes. However, we can make some generalizations. There appear to be two groupings of archaeal viruses with distinct evolutionary trajectories: first, those viruses that structurally resemble viruses that infect members of Eukarya and Bacteria (i.e., Caudovirales with relationships to herpes viruses of Eukarya and tailed bacteriophages), and a second grouping whose viruses only infect archaeal species. These latter viruses appear to have arisen independently from the other lineages of viruses and to have limited horizontal gene transfer with viruses infecting members of the other domains of life [1]. In order to gain a more in-depth understanding of archaeal virus evolution, we need to greatly expand our knowledge of viruses beyond the limited archaeal phyla that have been investigated to date.

## Looking forward

Detailed studies of viruses have often provided new insights into the biochemistry and cell biology of the hosts they infect. We fully expect that continued studies into archaeal viruses will be no exception. We are arguably in the “early days” of Archaeal virology, where future efforts to identify and culture archaeal viruses from across diverse archaeal phyla and to develop robust genetic, structural, and biochemical systems for their analysis will provide exciting discoveries into the archaeal lifestyle, expand our understanding of virus-host interactions, and provide new insights into the evolution of viruses and cells.
